# The Phosphatidylinositol 3-Kinase/mTor Pathway as a Therapeutic Target for Brain Aging and Neurodegeneration

**DOI:** 10.3390/ph4081070

**Published:** 2011-08-04

**Authors:** David Heras-Sandoval, Evangelina Avila-Muñoz, Clorinda Arias

**Affiliations:** Departamento de Medicina Genómica y Toxicología Ambiental, Instituto de Investigaciones Biomédicas, Universidad Nacional Autónoma de México, 04510, México D.F., Mexico; E-Mails: david.herassandoval@gmail.com (D.H.-S.); ediacaraart@yahoo.com.mx (E.A.-M.)

**Keywords:** PI3K, mTOR, insulin resistance, brain aging, Alzheimer's disease

## Abstract

Many pathological conditions are associated with phosphatidylinositol 3-kinase (PI3K) dysfunction, providing an incentive for the study of the effects of PI3K modulation in different aspects of diabetes, cancer, and aging. The PI3K/AKT/mTOR pathway is a key transducer of brain metabolic and mitogenic signals involved in neuronal proliferation, differentiation, and survival. In several models of neurodegenerative diseases associated with aging, the PI3K/AKT pathway has been found to be dysregulated, suggesting that two or more initiating events may trigger disease formation in an age-related manner. The search for chemical compounds able to modulate the activity of the PI3K/AKT/mTOR pathway is emerging as a potential therapeutic strategy for the treatment and/or prevention of some metabolic defects associated with brain aging. In the current review, we summarize some of the critical actions of PI3K in brain function as well as the evidence of its involvement in aging and Alzheimer's disease.

## 1. Introduction

The phosphatidylinositol 3-kinase (PI3K)/AKT serine/threonin protein kinase (AKT)/mammalian target of rapamycin kinase (mTOR) signaling pathway in central nervous system (CNS) has been implicated in metabolism regulation and energy homeostasis [1] and has emerged as an important mediator of neuronal physiology, appearing to serve as a direct link between aspects of learning and memory, neuronal survival, neurogenesis and apoptosis [2,3]. The PI3K is highly conserved through evolution, and has been described in species ranging from insects to mammals [3]. PI3K signaling has been implicated in aging and lifespan regulation, and the proliferation of adult neuronal progenitor cells, as well as in synaptic plasticity, which gives PI3K relevance to cognitive processes in addition to pathological brain aging and neurodegeneration [1-8]. The ability of PI3K to control key functions of the cell provides the incentive for investigations into the effects of the modulation of PI3K signaling pathways in different aspects of neuronal physiology, including its role in neuronal development as well as brain aging and dysfunction.

The PI3Ks are multifaceted enzymes that are primarily responsible for the production of 3′ phosphoinositides from phosphatidylinositol in response to growth factors. The most common PI3K is PI3K IA, a functional heterodimer comprised of two subunits, one catalytic and one regulatory, with molecular weights of 110 kD (p110) and 85 kD (p85), respectively [4,9,10]. Three different genes (p85α, p85β, and p55γ) encode the regulatory unit. In addition, the p85α mRNA can undergo alternative splicing to produce p50α and p50α/AS53. The p110 protein exists as one of four isoforms (α, β, γ, δ) [1].

Most neurons express all the isoforms of the regulatory p85 subunit, but relatively high levels of the catalytic p110α are found in the hippocampus, olfactory bulb, and cerebellum [2,11]. The various isoforms can result in different roles for PI3K in cellular physiology depending upon the sub-cellular location, the metabolic cellular context, and the involvement of upstream signals and receptor signaling pathways [3].

## 2. The PI3K/AKT Pathway in Neuronal Plasticity

The PI3K/AKT pathway is a key transducer of mitogenic and metabolic signals that drive proliferation and inhibit both the apoptosis and differentiation of adult neuronal progenitors [12]. Activation of PI3K is induced by many growth factors such as insulin and insulin like growth factor 1 (IGF1), Ras proteins, or the diminished activity of the phosphatase and tensin homolog (PTEN). The activation of the catalytic subunit of PI3K leads to the phosphorylation of phosphatydylinositol-4,5-bisphosphate (PIP2) to generate phosphatidylinositol-3,4,5-trisphosphate (PIP3). In turn, PIP3 drives the activation of the phosphoinositide-dependent protein kinase-1 (PDK-1). The serine-threonine kinase, AKT, is recruited to the cell membrane and phosphorylated by PDK-1, which stimulates the catalytic activity of AKT, which in turn phosphorylates and downregulates glycogen synthase kinase 3 β (GSK3β). Once activated, AKT is able to phosphorylate numerous downstream signaling proteins, including members of the Bad-dependent apoptosis machinery, the forkhead transcription factor proteins (FOXOs), the nuclear factor κB transcription factor (NFκB) and GSK3β, as well as mTOR, which modulates protein translation machinery [12]. In addition, signaling proteins that contain pleckstrin homology (PH) domains accumulate at sites of PI3K activation in the inner surface of the plasma membrane *via* an interaction between the PH domains and the phospholipid products of PI3K [4,5,12].

During brain development, PI3K is involved in a variety of cellular functions that include proliferation, cell migration, and axon guidance [13]. In addition, PI3K activity is essential for microtubule transport during axonal growth cone formation and for the establishment of neuronal polarity [16]. Signaling of PI3K/AKT *via* the cAMP response element-binding (CREB) transcription factor is involved in the proliferation of hippocampal neuronal cells following fibroblast growth factor 2 (FGF2) stimulus and activation of the FGF receptor 1 [12]. In adult brain, the activation of PI3K promotes the survival of newly-formed dentate gyrus granule cells generated during exercise, which lead to increase in synaptic plasticity [14].

**Figure 1:**
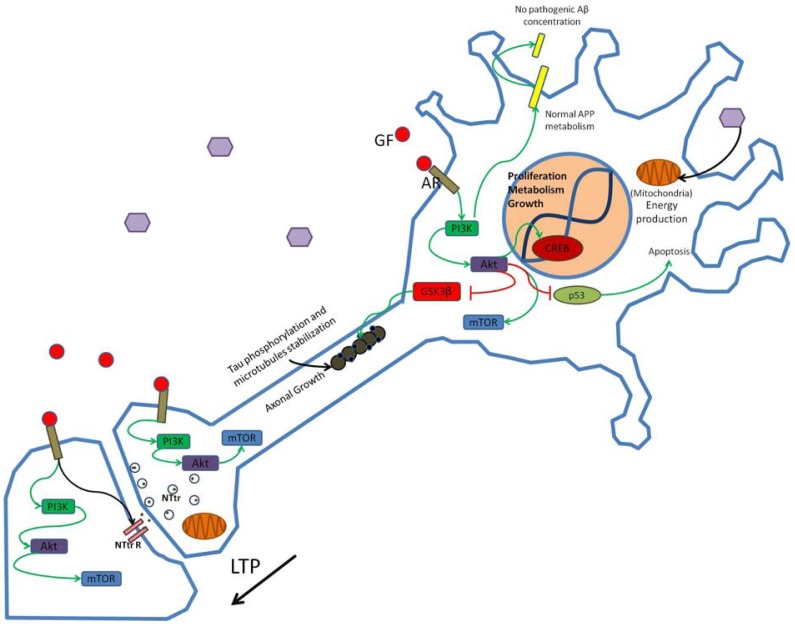
PI3K signaling in normal neural function and LTP induction.

The role of the PI3K/AKT signaling cascade in synaptic plasticity and memory processes in the mature brain deserves a special mention. In the adult brain, evidence has accumulated for a variety of mechanisms by which PI3K signaling modulates long-term potentiation (LTP), a cellular correlate of memory. Intra-cerebro-ventricular (i.c.v.) infusions of the PI3K inhibitor LY294002 blocked LTP maintenance in the *in vivo* hippocampus and were associated with a transient phosphorylation of the PI3K substrate AKT at Ser473 [15]. Additionally, *in vivo* infusion of the PI3K inhibitor wortmannin together with LY294002 and the mTOR inhibitor rapamycin into the pre-frontal cortex also inhibited the long-term retention of trace fear memory [16]. PI3K may influence synaptic plasticity *via* the modulation of receptor trafficking to the synaptic membrane, a mechanism that promotes rapid and long lasting synaptic changes [17,18]. AKT is also involved in the control of synaptic strength, *via* phosphorylation of the GABA receptor. In this regard, it is possible that insulin stimulation induces a localized activation of PI3K at dendritic synapses, which in turn induces activation of AKT in the post-synaptic vicinity [19]. Interestingly, exposure to neurotrophin 3 (NT-3) or brain derived neurotrophic factor (BDNF) produces synapse maturation at the neuromuscular junction (NMJ) as well as dendritic sprouting in hippocampal neurons, and in both kinds of neurons, PI3K signaling promotes long-lasting changes in synaptic strength that depend upon both presynaptic and postsynaptic protein synthesis mediated *via* the activity of the eukaryotic translation initiation factor alpha (eIF2-α), a target of mTOR [20,21] (Figure 1).

It has been suggested that signaling pathways that play a relevant role during development may also be implicated in aging [22]. Accordingly, mutations that inactivate certain signaling pathways, such as insulin/PI3K signaling, slow senescence and increases lifespan up to several folds in mouse models [23].

## 3. PI3K and Brain Aging

Brain aging is a highly complex process involving multiple systems and cellular pathways. Since more than a decade it is known that mutation in some elements of the insulin/PI3K pathway significantly impact longevity [7,24]. A constant feature of aging is the induction of stress response pathways controlled at the molecular level by a number of highly conserved molecules and transcriptional regulators, including proteins of the insulin and insulin like growth factor 1 (IGF1), mTOR, sirtuin system, and AMP activated protein kinase (AMPK) pathways [26-28].

The search for longevity-related genes has led to the increased understanding of signaling pathways involved in the regulation of lifespan in some model organisms. Among these, insulin/PI3K signaling has been demonstrated to be a key player in the control of longevity. Insulin, as well as insulin like growth factor 1 (IGF1), binds to the insulin receptor (IR). Adapter proteins, such as insulin receptor substrate proteins (IRS1-4), bind to tyrosine residues and, in turn, activate PI3K and AKT, which then act upon different target proteins such as GSK3β and mTOR [7,8].

Mutations in some genes that participate in PI3K signaling can lead to increased life span. In *Caenorhabditis elegans* (*C. elegans*), mutations in Age-1, a PI3K catalytic subunit homologue, produce an elevated lifespan [29-31]. Another example is Daf-2, an IR homologue, a temperature-dependent mutation of which nearly doubles lifespan in *C. elegans*. Daf-2 produces stress resistance and longevity *via* the inhibition of the FOXO transcription factor homologue Daf-16. In addition, loss of the *Drosophila melanogaster* IRS homologue, CHICO, has shown to increase longevity [31].

In mammals, the role of the insulin/IGF1/PI3K pathway in longevity seems to be more complex. For example, in older humans, a compensatory hyperinsulinemia is developed to maintain glucose homeostasis and prevent type 2 diabetes mellitus (T2DM). Nevertheless, elevated levels of circulating insulin have negative effects on the brain and can diminish lifespan. It has been proposed, therefore, that attenuating insulin signaling in aged or in overweight people may halt the damaging effects of hyperinsulinemia [32]. In support of this idea, mice with IR mutations were found to have diminished adipose tissue and increased longevity [33]. Interestingly, the selective mutation of brain IRS2 is sufficient for increased longevity in mice, suggesting that targeted inhibition of this protein may also impact longevity in humans [32]. Although the role of insulin signaling in human longevity is controversial, it has been shown that centenarian individuals show an increase in peripheral sensitivity to insulin and reduced levels of circulating insulin [34]. Findings from studies of nonagenarians likewise suggest that longevity may be associated with differences in insulin sensitivity [35]. Two external factors that impact aging are known to be physical exercise and caloric restriction; this last factor, in particular, appears to have important effects on longevity-related processes. Caloric restriction in mice reduces the intensity and duration of insulin secretion required for glucose homeostasis, leading to diminished insulin levels and promoting the expression of antioxidant enzymes [36].

The mTOR kinase function, a downstream participant of the insulin/PI3K pathway, may be particularly important for longevity. Numerous cellular signals regulate mTOR activity; these include low oxygen pressure, reduced nutrient concentration, oxidative stress, and DNA damage. Once activated, mTOR participates in nutrient importation, translation of messenger RNA, and the biogenesis of ribosomes. mTOR activates the eukaryotic translation elongation factor 2 (eEF2), eEF2 kinase (eEF2K), ribosomal protein S6 kinase (S6K), which regulates protein synthesis and gene transcription [37,38]. Diminished activation of the PI3K/AKT/mTOR pathway significantly augments longevity in mice [39]. Likewise, centenarian individuals that show increased sensitivity to insulin also have decreased mTOR activity. Thus, longevity seems to be associated with the reduced activity of the insulin or IGF-mediated PI3K/AKT/mTOR pathways, implicating these signaling cascades as important targets for pharmacological manipulation [22]. Importantly, mTOR activates S6K, which in turn phosphorylates IRS proteins in serine residues, inhibiting insulin signaling, by a negative feedback loop of regulation [40]. The PI3K/mTOR pathway also plays an important role in autophagy, a catabolic process that helps to maintain cellular homeostasis. The regulation of autophagy is intimately associated with the control of cell growth, cell proliferation, cell survival, and cell death. Impairments of autophagy have also been demonstrated in neurodegenerative diseases, such as Alzheimer's disease (AD) [40,41].

Along PI3K/AKT/mTOR pathway the sirtuin system, found from bacteria to mammals, has shown to play an important role in controlling longevity, oxidative stress, insulin resistance, metabolism and neuroprotection. Sirtuins belongs to a family of class III histone deacetylases that transfers acetyl groups from lysine residues to ADP-ribose moiety of NAD^+^ producing a deacetylated protein, nicotinamide and a 2′-O-acetyl-ADP-ribose. SIRT1 and SIRT2 are highly expressed in brain [27,42-44]. It has been probed that SIRT1 interacts directly with the p85 subunit of PI3K forming a complex that, after insulin stimulation, binds to IRS1/2 and activates this pathway. Moreover, SIRT1 levels correlate positively with AKT phosphorylation at serine 473. Other target proteins of SIRT1 are AMPK, acetyl CoA synthetase, glutamate dehydrogenase, IRS, histones, and other molecules involved in the modulation of energy metabolism, stress responses, and cell survival [27,42-45].

**Figure 2:**
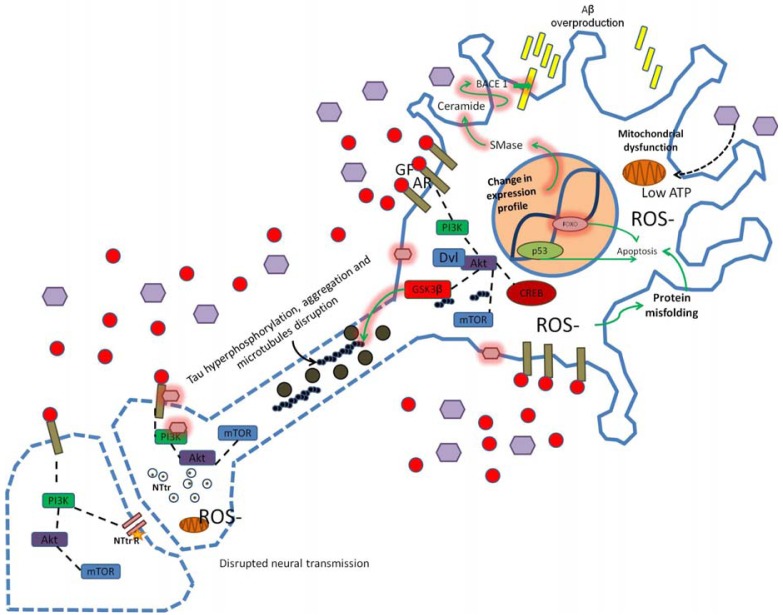
PI3K signaling in neural aging and disease.

Biological processes linked to normal aging also include systemic inflammatory and immune responses, oxidative stress, and altered calcium regulation. Among these, oxidative stress leads to mitochondrial dysfunction resulting in reduced respiratory metabolism and the increased generation of reactive oxygen species (ROS). Also, accumulation of DNA damage may results from both increased oxidative damage and the reduced efficiency of DNA repair, predisposing the cell to apoptosis, senescence, and inflammation. Aging is also associated with protein misfolding and subsequent aggregation in the cytoplasm, nucleus, and endoplasmic reticulum. Age-related cellular damage, as well as stress and physiological decline, contribute to the pathogenesis of age-related diseases including metabolic syndromes, inflammatory disorders, cancer, and neurodegenerative diseases [46-49] (Figure 2).

### 3.1. Oxidative Stress and PI3K in Aging

ROS are produced at low levels during normal physiological conditions and are scavenged by endogenous antioxidant systems that include superoxide dismutase (SOD), glutathione peroxidase, catalase, and vitamins E and C [50]. When the generation of ROS overrides the scavenging ability of the endogenous antioxidant system, oxidative stress occurs in the cell. Several features of the brain suggest that it is highly sensitive to oxidative stress that, combined with preexisting impairments in metabolic status, leads to the damage and destruction of both neural and vascular cells. Aging is accompanied by a general increase in oxidative stress, perhaps due to decreased in antioxidant defenses [51-53].

AKT is a critical survival factor that can modulate cellular pathways in both the central and peripheral nervous systems. Activation of AKT is an essential PI3K-dependent regulatory step in the cellular response to oxidative stress. Early studies have demonstrated that over-expression of AKT in neurons prevents apoptosis during growth factor withdrawal [54]. CNS expression of AKT1 and AKT2 are increased in the early stages of embryonic development, but decreases gradually in post-natal cells [49]. In the adult brain, the expression of AKT1 and AKT2 is weak; however, a dramatic increase in the expression of AKT1 mRNA and protein is induced when cells are subjected to injury [55-57]. AKT expression has shown to be necessary and sufficient for neuronal survival, as the expression of dominant-negative AKT, or the pharmacological inhibition of PI3K, causes neurons to undergo apoptosis even in the presence of neurotrophic factors [58] and induces cell death during oxidative stress [57,59]. Further studies have confirmed that endogenous cellular stores of AKT provide the cell protection from injury [56,57,59]. In diverse paradigms of neuroprotection, AKT phosphorylation mediates neuronal survival elicited by antioxidants [60]. Oxidative stress associated with high cholesterol intake impairs insulin signaling, increases serine phosphorylation of IRS1, and suppresses insulin-stimulated PI3K and AKT activities, leading to increased stress activated c-Jun N-terminal kinase (JNK) activity that underlies cognitive impairments in mice [61]. Reciprocally, the activity of mTOR may increase the mitochondrial production of ROS by activating mitochondrial respiratory capacity [62].

Thus, a proper balance between the transient and sustained activation of PI3K/AKT seems to be important for neuronal survival under diverse circumstances associated with cell injury and oxidative stress.

### 3.2. PI3K Signaling and Alzheimer's disease

AD is the most prevalent progressive neurological disease in the elderly population, affecting millions of individuals throughout the world. One of the two main pathological hallmarks of AD is the deposition of amyloid β protein (Aβ) in the form of senile plaques throughout the hippocampus and neocortex [63]. The accumulation of Aβ appears to be influenced by many complex processes including multiple proteolytic events involved in Aβ production and defects in Aβ degradation and removal. Although the pathophysiology of AD is matter of intense study, a promising area of research concerns the elucidation of abnormal spatio-temporal integration of metabolic signals, particularly those downstream of PI3K/AKT/mTOR activity. Haugabook *et al.* demonstrated a significant reduction (40–50%) in Aβ accumulation in the Tg2576 transgenic mouse model of AD after oral administration of the PI3K inhibitor wortmannin. In addition, it was found that wortmannin may influence the trafficking of the amyloid precursor protein (APP) and/or its metabolites, resulting in a decreased secretion of the Aβ peptide [64]. In line with the above evidence, it has been found a bidirectional modulation of the APP metabolism by insulin in neuroblastoma cells: increasing the release of soluble APP [65], reducing intracellular levels of Aβ40/42 and increasing Aβ40/42 secretion [66]. Additional evidence shows that PI3K mediates a switch in the expression of neurotrophin receptors, from the high affinity catalytic neurotrophin receptor that binds specifically nerve growth factor, TrkA, to the low affinity neurotrophin receptor, p75NTR, which favors sphingomyelinase activity, ceramide production and the stabilization of β-site of the APP cleaving enzyme (BACE1) suggesting that Aβ production depends on growth factors and the cell metabolic state [67]. Insulin-mediated PI3K/AKT signaling also regulates the phosphorylation and cellular relocalization of presenilin 1, a γ-secretase complex enzyme, which in turn may increase Aβ production [68] (Figure 2).

In addition to the role of PI3K signaling in Aβ generation, PI3K activity also contributes to the expression of biochemical alterations in the tau protein, which contains a consensus motif for AKT phosphorylation. The tau motif includes the AT100 double phospho-epitope (Thr212/Ser214), which is a specific marker for AD and other neurodegenerative tauopathies, and which also may play a specific role in AKT-mediated anti-apoptotic signaling [69]. Several studies have shown tau phosphorylation in rodents after exposure to different stressors such as food deprivation [70] or forced swimming [71]. This raises the possibility that tau phosphorylation is an integral part of the neuronal response to stressors, and that PI3K/AKT is a part of this response. On the other hand, PI3K inhibition with wortmannin leads to GSK3β activation, which in turn increases tau phosphorylation at specific epitopes that may contribute to the paired helicoidal filaments (PHF) formation in cortical and hippocampal neurons. This effect is reversed upon inhibition of GSK3β with lithium chloride [72]. Thus, decreased growth factor stimulation of PI3K signaling in aging or disease may allow increased GSK3β activity, leading to tau hyperphosphorylation. Interestingly, it was found that p-AKT levels and activity are decreased in AD brain specimens and that Aβ interferes with AKT activation [73], which could be involved in the increase of GSK3β activity and tau phosphorylation.

Taking into account AD complexity and the dual role of PI3K signaling in the development of markers for AD it becomes clear that an understanding of the complex interaction between insulin signaling and insulin resistance is necessary for the development of novel drug therapies for the treatment and/or prevention of this neurodegenerative disease.

### 3.3. Insulin Resistance in Aging and AD

Insulin resistance associated with T2DM and obesity increases with aging and represents a risk for the development of cognitive deficiencies such as low perceptual speed and, in some cases, AD [74-77]. T2DM and insulin resistance are closely associated with obesity, dyslipidemia, high blood pressure, and pro-thrombotic and pro-inflammatory states. Together these factors constitute the metabolic syndrome [78]. Insulin resistance is a major pathological condition, and is often co-morbid with elevated blood pressure, cardiovascular disease, dyslipidemia, and high cholesterol levels. T2DM has been identified as a risk factor for AD, with increasing epidemiological evidence showing that T2DM almost doubles the risk of developing AD, and this risk increases when associated with cardiovascular disorders or dyslipidemia [75-77,79,80].

Aging is associated with low levels of insulin and insulin receptors in brain. Diminished cerebral insulin levels and peripheral insulin resistance appears to be accompanied with disturbances in insulin signaling in AD [81], that has led many to consider this neurodegenerative disease as an insulin-resistant brain condition [82]. The relationship between AD and T2DM has been explored regarding central and peripheral insulin signaling, for example, in a Japanese epidemiological study demonstrating that high insulin and/or high glucose plasma levels in T2DM patients accelerate amyloid load and amyloid plaque formation in individuals with the APOE4 allele [67,77,80].

Several lines of evidence suggest that hyperglycemia is associated with cognitive impairment and with structural alterations in the brain [83,84]. Prolonged hyperglycemia, dyslipidemia and oxidative stress in diabetes, result in the increased production and accumulation of advanced glycation end products (AGEs) [80,85-88]. In addition, it was demonstrated that oxidative stress leads to activation of FOXO transcription factors through PI3K/AKT signaling pathway [89].

As mentioned, another remarkable feature of insulin action in brain is the regulation of APP metabolism. High insulin levels diminish Aβ clearance, perhaps by competing for the insulin-degrading enzyme (IDE). On the other hand IDE expression is downregulated in hyperinsulinemic conditions, and is considerably diminished in AD patients with T2DM [80]. Insulin/IGF1 signaling protects synaptic dendrites from Aβ oligomers injury [90].

The relevance of the insulin/PI3K pathway to the development of AD biomarkers has been studied in a mouse model of hyperinsulinemia in which systemic insulin administration promoted tau phosphorylation as shortly as 10 min after insulin administration [91]. In this sense, it has been demonstrated that insulin prevents the phosphorylation of presenilin 1 *via* PI3K/AKT and in this way regulates Aβ production [92]. Aβ oligomers-treated neurons exhibit elevated levels of activated PI3K, AKT and mTOR and AKT or mTOR inhibitors blocked Aβ oligomers-induced aberrant neuronal cell cycle reactivation [93]. Finally, some studies in mice lacking the neuron-specific insulin receptor have shown a complete loss of insulin-mediated activation resulting in markedly reduced phosphorylation of AKT and GSK3β, leading to substantially increased phosphorylation of tau a hallmark of AD [94].

Liu *et al.*, have investigated the insulin/PI3K/AKT signaling pathway in the autopsied frontal cortices from AD, T2DM, T2DM–AD and control cases. They found decreased levels and activities of several components of this pathway in patients with T2DM and AD (T2DM-AD) [95]. It is of interest the recent finding that the hormone glucagon-like peptide-1 (GLP-1) facilitates insulin signaling, opening the possibility of using analogs of this hormone to improve cognition in AD [96].

A rat model was developed by using streptozotocin (STZ) to induce the brain insulin system dysfunction. STZ is a drug that selectively destroys insulin-secreting pancreatic β cells and thereby causes type 1 diabetes mellitus (T1DM) [97]. Insulin deficiency after systemic STZ administration reproduces some aspects of the AD-like pathology such as tau hyperphosphorylation and a reduced phosphatase activity [98-102]. Although STZ icv does not cause systemic DM it induces alterations in brain metabolic pathways being under control of the insulin signaling found in the AD's brain and aggravates the expression of AD markers in transgenic AβPP-overexpressing mice *via* GSK3 pathway [103]. STZ is a toxic glucose analogue that diminishes ATP content, and eventually produces oxidative stress, DNA fragmentation, myelin neurotoxicity, low synthesis of acetylcholine, and cognitive impairment [97,104,106,107]. The uptake of STZ uses the glucose transporter 2 (GLUT2) to enter cells and for this reason hypothalamic neurons are particularly vulnerable to STZ [105].

## 4. How Can the Modulation of PI3K Signaling Improve Neuronal Dysfunction in Aging and Disease?

In this review, we have described how aging is associated with changes in several transduction pathways. In particular, we have reviewed how insulin/IGF1-dependent PI3K/AKT/mTOR dysregulation is associated with abnormal cellular functions that lead to cellular stress, cellular senescence, altered neuronal plasticity, apoptosis, and cell death. The hippocampus is a nodal region in the control of neuronal reactions to stress, and is highly plastic region implicated in spatial learning and memory, which make the hippocampal circuit a particularly vulnerable brain site. Therefore, research involving PI3K modulation in this specific brain region may help to retard senescence and/or aging-related brain damage and cognitive impairments. However, the pleiotropic nature of the PI3K/AKT pathway makes its selective inhibition/activation difficult. The analysis of specific PI3K isoforms and the development of specific molecular modulators are necessary to address this problem. In this regard, there is currently work being conducted into the structural analysis of oncogenic PI3Kα mutations as a basis for the molecular design of isozyme-specific and mutation-specific inhibitors for individualized cancer therapies [108].

There is the possibility of ameliorating some of the molecular hallmarks of AD. For example, it has been reported that the PI3K inhibitor LY294002 induces the accumulation of a highly ubiquitinated form of presenilin-1. Although the biological significance of this is not known, possible interventions into PI3K-related Aβ generation therefore exist [109]. Interestingly, a recent trial with the insulin sensitizer, rosiglitazone, showed positive trends in mild-to-moderate AD patients [110].

Another important candidate for the modulation of PI3K signaling is mTOR, the downstream PI3K target. For example, inhibition of mTOR activity regulates longevity by stimulating cellular autophagy and the removal of misfolded proteins and damaged organelles, thus restoring cellular function [37,38,111]. It is interesting to note that hyperactivation of mTOR is associated with multiple degenerative diseases, autoimmune diseases, and cancer, therefore its inhibition is a reasonable strategy to combat such disorders [37].

The main mTOR inhibitor, rapamycin, is well-tolerated by humans and is interesting as an anti-aging drug. Nevertheless, it is necessary to clearly understand the role of mTOR action in normal cell functioning [37,112,113]. Rapamycin has been proposed for the treatment of neurodegenerative disorders, as it is a hydrophobic molecule that readily crosses the blood-brain barrier. Rapamycin promotes autophagy, and therefore may combat protein aggregates like tau or Aβ [37]. Recently, novel catalytic inhibitors of mTOR have been designed that are synthetic small molecules that function as competitive ATP-binding inhibitors [114,115]. In addition, newly developed dual mTOR-PI3K inhibitors have been described that counteracts the PI3K/AKT overstimulation that occurs when mTOR alone is inhibited [116,117].

Another candidate to be regulated is the sirtuin system implicated in insulin/PI3K/AKT/mTOR pathway modulation. Several conditions such as caloric restriction, and exercise as well as the compound resveratrol improve insulin/PI3K/AKT/mTOR signaling pathway trough modulation of sirtuin content [27,42-45].

## 5. Conclusions

The modulation of molecules involved in signal transduction pathways is an emerging therapeutic option for a number of human diseases. The PI3K/AKT/mTOR signaling pathway coordinates a variety of complex events that lead to changes in cell metabolism, cell growth, cell movement, and cell survival. Further studies of this pathway should be directed towards the design of new small-molecule modulators of PI3K isoforms implicated in neurodegeneration.

## Figures and Tables

Growth factors (GF) such as insulin bind to appropriate receptors (AR) and activate the PI3K pathway modulating synaptic structure, function and neural transmission. Through phosphorylation of enzymes and enhanced gene transcription PI3K activation promotes neuronal growth and survival and participates in the establishment of long-term potentiation (LTP). Hexagons represent normal glucose levels and utilization for energy production. Schematic representation of non-pathogenic amyloid precursor protein (APP) metabolism and tau function in neurons is shown. NTtr, neurotransmitter; NTtrR, neurotransmitter receptor; p53, transcriptional regulatory protein 53.

Alterations in PI3K signaling due to oxidative stress, insulin resistance, hyperinsulinemia, hyperglycemia, high cholesterol or inflammation alter synaptic structure, function and neural transmission. Modification in the APP/Aβ metabolism leads to Aβ overproduction and dysregulation of GSK3 activity may induce tau hyperphosphorylation. Changes in cellular microenvironment leads to elevated ROS production, mitochondrial dysfunction, low ATP levels and increased neuronal damage (as indicated by dotted blue lines in axonal and synaptic regions of the neuron scheme). Star represents low neurotransmitter receptor levels, purple hexagons represent high glucose concentration, pink hexagons represent chemical modification of proteins/membrane (e.g., glycosylation), GF, growth factor; AR, appropriate receptor; Dvl, disheveled protein; SMase, sphingomyelinase.
